# Esophagogastroduodenoscopy Outcomes Variated by the Time of the Day: A Single-Center Experience

**DOI:** 10.3390/jcm12030863

**Published:** 2023-01-21

**Authors:** Zhang Zhang, Xiaojia Chen, Haizhou Wang, Haihang Nie, Fan Wang, Qiu Zhao, Jun Fang

**Affiliations:** Department of Gastroenterology, Zhongnan Hospital of Wuhan University, Wuhan 430071, China

**Keywords:** esophagogastroduodenoscopy, quality indicator, time factors

## Abstract

(1) Background: To assess whether the start time influences the outcomes of esophagogastroduodenoscopy (EGD). (2) Methods: We retrospectively analyzed the clinical data of patients who underwent EGD between January 2021 and December 2021 in our endoscopy center. The EGD were divided into three shifts, according to the start time. The lesion detection rate (LDR) and endoscopy biopsy rate (EBR) were used to evaluate the quality of the EGD. (3) Results: A total of 14,597 procedures were included in this study. The LDR of shift 2 was significantly lower than that of shift 1 (62.4% vs. 58.5%; *p* < 0.001). The EBR of shift 1 (37.4% vs. 31.5%; *p* < 0.001) and shift 3 (35.5% vs. 31.5%; *p* = 0.024) were significantly higher than that of shift 2; the EBR in shift 1 did not differ significantly from shift 3 (*p* = 0.280). The multivariable analysis for the EGD performed before 14:00 demonstrated a graded decrease in the LDR and EBR after adjusting the confounders (*p* < 0.001). (4) Conclusion: In a continuous working period, the lesion detection and biopsy submission of EGD are superior to those in the first three hours compared to the last three hours; the LDR and EBR decreased as the day progressed, probably due to the endoscopists’ fatigue.

## 1. Introduction

According to the GLOBOCAN 2020 estimates, gastric cancer and esophageal cancer were still among the top ten most diagnosed cancer types and the leading causes of cancer-related death worldwide [1]. Geographically, Asian regions exhibited the highest morbidity for esophageal and gastric cancers [2]. As a result of economic progress and dietary improvements, the incidence of the two cancers continued to decline. However, there an increasing incidence of gastric cancer was seen among young populations (<50 y) in low-risk and high-risk countries [3]. The endoscopic screening proved critical in the prevention of upper gastrointestinal (UGI) cancer. Early detection and screening for precancerous lesions represent an effective intervention to reduce the health care burden through the removal of precancerous lesions and interrupting their progression to invasive phases of the disease. Endoscopic screening programs were found to contribute to decreasing the gastric cancer-related mortality in many cohorts [4,5,6]. Therefore, esophagogastroduodenoscopy (EGD) has been widely used for esophageal and gastric cancer screening.

However, early cancer and lesions are often subtle and are rarely recognized during EGD examination. Several studies have estimated that a significant minority of esophageal and gastric cancers are missed by endoscopy [7,8]. A high-quality EGD ensures a precise examination of the UGI mucosa. Thus, EGD quality control is critical for ensuring efficacy, which helps to increase the recognition of lesions and reduce the incidence of UGI cancer. A robust indicator is crucial for the evaluation of EGD quality, in contrast to colonoscopy, where several performance measures (withdrawal time, adenoma detection rate [ADR], and cecal intubation rate) have been well-identified [9]. The British Society of Gastroenterology (BSG) and the European Society of Gastrointestinal Endoscopy (ESGE) have developed guidelines to improve EGD quality [10,11], including performance metrics such as pre-, intra-, and post-procedural activities. However, with the exception of inspection time, there was a lack of measurable quality indicators such as ADR and cecal intubation rate. Therefore, many studies have focused on finding convenient and effective surrogate parameters. Januszewicz et al. used the endoscopy biopsy rate (EBR) as a quality indicator. They confirmed that EBR was associated with gastric premalignant condition detections and missed gastric cancer rate [12]. Roman’czyk et al. proposed a composite detection rate (CDR), which was defined as the proportion of patients in whom at least one of the following lesions were detected: esophageal inlet patches, gastric polyp, and post-ulcer duodenal bulb deformation. The indicator was validated for the detection of UGI neoplasms [13].

Several studies have demonstrated that the start time can affect colonoscopy and endoscopic retrograde cholangiopancreatography (ERCP) outcomes [14,15,16,17]. Fatigue or abstraction may contribute to the decrease in the endoscopy outcomes. A previous study has examined the impact of the procedure time and queue position on the sensitivity and diagnostic accuracy of endoscopic ultrasound-guided fine-needle aspiration (EUS-FNA) for pancreatic lesions [18]. According to one study, the time of day had no effect on the diagnostic and therapeutic efficacy of small-bowel enteroscopy. Some studies have observed that morning colonoscopies had higher ADR than afternoon colonoscopies and considered the time of day to be an independent factor. [19] However, the time variable has not been previously examined in relation to EGD outcomes. We believe that an increased diagnostic yield is associated with an increased EGD quality. Thus, the lesion detection rate (LDR) and EBR were used as quality indicators of EGD. The study aimed to explore the data of the endoscopy center to assess whether the start time influences EGD outcomes.

## 2. Materials and Methods

A retrospective study was carried out on outpatients who underwent diagnostic EGD in the endoscopy center of Zhongnan Hospital of Wuhan University between January 2021 and December 2021. The exclusion criteria were: (i) age < 18 years; (ii) inpatients and emergency patients; and (iii) incomplete data of interest. The recorded parameters were: (i) patient demographics: sex, and age; (ii) procedure information: start time, conscious sedation, name of the endoscopist, sex of the endoscopist, and biopsies taken; (iii) endoscopy findings: Barrett’s esophagus, submucosal lesion, polyp, intestinal metaplasia, Helicobacter pylori infection, ulcer, cancer, atypical hyperplasia, reflux esophagitis, and others. Gastritis and gastric atrophy were regarded as negative outcomes because they are easily diagnosed and shared in the Chinese population.

Patients undergoing EGD should schedule at least one day in advance, and the nurse will schedule the operation time. The EGD appointment time is scheduled according to the patient’s preference, without the influence of either patient symptoms or the need for conscious sedation. All of the EGD were performed by gastroenterologists or internal medicine specialists who have received complete endoscopic training. Endoscopists were assigned a half-day block. The endoscopists in the morning shift performed endoscopies from 8:00 to 13:59. The endoscopies after 14:00 were performed by endoscopists scheduled in the afternoon block. Our center requires at least seven minutes of inspection time. The start time of each endoscopy was evaluated in 1 h intervals. According to the start time, the procedures were divided into shift 1 (08:00–10:59), shift 2 (11:00–13:59), and shift 3 (after 14:00). Written informed consent was signed before each EGD procedure.

The LDR was calculated as the proportion of EGD in which at least one of the aforementioned lesions were detected. The EBR was defined as the proportion of EGD with at least one biopsy specimen obtained for histology from the esophagus, stomach, or duodenum. The primary outcome of this study was to assess the influence of the start time on EGD outcomes. The second outcome was to explore the factors affecting the LDR and EBR and the relationship between the two quality metrics. The study protocol was approved by the Ethics Committee of Zhongnan Hospital of Wuhan University [approval number 2020110].

## 3. Statistical Analysis

The quantitative variables were presented as the median and interquartile range (IQR). Numbers and percentages were used to present qualitative variables. A Wilcoxon-Mann-Whitney U test was used to compare the continuous variables. A chi-squared test was used to compare the categorical variables. Tests for the LDR and EBR between groups were performed using the chi-squared test. Bonferroni correction was used for pairwise comparison between groups. Binary logistic regression was used to calculate the unadjusted odds ratios (ORs) and 95% confidence intervals. The mantel-Haenszel test was performed to analyze the influence of time on the LDR and EBR. Univariate and multivariate analyses were utilized to evaluate whether the start time can predict the LDR and EBR. Spearman correlation was carried out to measure the association between the EBR and LDR. All of the analyses were performed using the Statistical Package for the Social Sciences, version 23.0 (SPSS, Chicago, IL, USA). A *p* value < 0.05 indicated statistical significance, and a *p* value < 0.0167 was considered as statistically significant for the Bonferroni correction.

## 4. Results

### 4.1. Baseline Characteristics

Between January 2021 and December 2021, 20,057 EGD were performed by 30 endoscopists in our endoscopy center. After applying the exclusion criteria, 14,597 cases were included; these were subcategorized into three groups based on their start time. Table 1 compares the three time periods according to the risk factors and EGD outcomes. Of these, 10,208 (69.9%) cases were performed in shift 1, 3541 (24.3%) cases were performed in shift 2, and 848 (5.8%) cases were performed in shift 3. The median age of patients was 49 (30–58) years, and 7666 (52.5%) were female. A total of 10,533 (72.2%) EGD were performed under conscious sedation. A total of 7920 (54.3%) cases were performed by female endoscopists. The comparison of the EGD findings are presented in Table 2. The detection rate of each kind of lesion in shift 1 and shift 3 was higher than that of shift 2.

### 4.2. Detection Rate and Biopsy Rate

**Figure 1:**
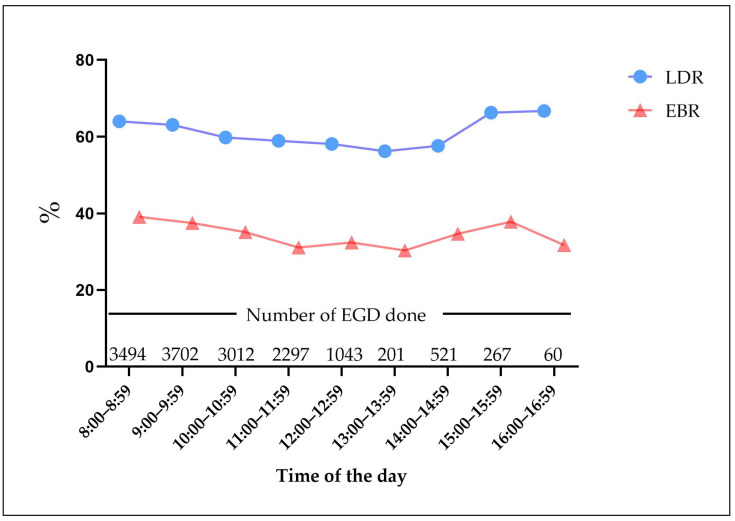
LDR and EBR as time progressed. The number of EGD corresponding to each hour denotes the total number of EGD conducted during that hour. EGD, Esophagogastroduodenoscopy; LDR, lesion detection rate; EBR, endoscopy biopsy rate.

**Figure 2:**
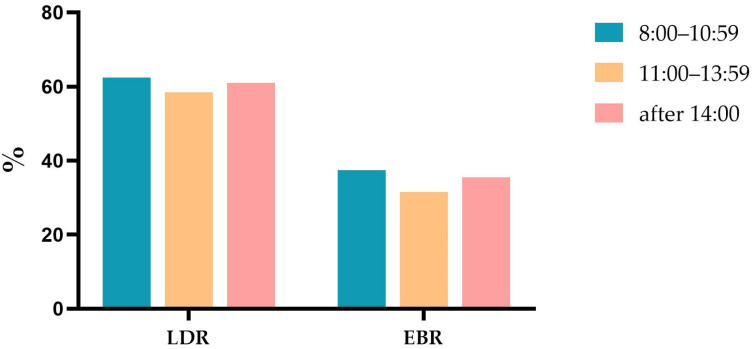
The comparison of LDR and EBR in three shifts. LDR, lesion detection rate; EBR, endoscopy biopsy rate.

The LDR was 61.4% and the EBR was 35.8% for all of the procedures. Figure 1 depicts the hourly trend in the detection and biopsy rate. A graded decrease in the LDR (*p* < 0.001) and EBR was (*p* < 0.001) observed for the EGD carried out before 14:00. The maximal detection rate was 64.0% in the first hour of the morning, while the lowest LDR was 30.3% between 13:00–13:59. Similarly, the maximal EBR of 39.1% was identified between 8:00–8:59, while the lowest EBR of 30.3% was found in the last hour. Compared with the previous EGD, the trend of the LDR and EBR showed a significant rise after 14:00. Figure 2 depicts the comparison of the detection and biopsy rates among the three shifts. Both the LDR (62.4% vs. 58.5%; OR = 0.848; 95% CI: 0.785–0.917; *p* < 0.001) and the EBR (37.4% vs. 31.5%; OR = 1.300; 95% CI:1.198–1.410; *p* <0.001) in shift 1 were higher than in shift 2. There was no significant difference in the LDR and EBR between shift 1 (*p* = 0.395) and shift 3 (*p* = 0.280). The LDR (58.5% vs. 61.0%; OR = 1.107; 95 % CI:0.950–1.291; *p* = 0.192) and the EBR (31.5% vs. 35.5%; OR = 1.199; 95 % CI:1.024–1.403; *p* = 0.024) were lower in shift 2 than shift 3. However, there were no statistically significant differences.

**Figure 3:**
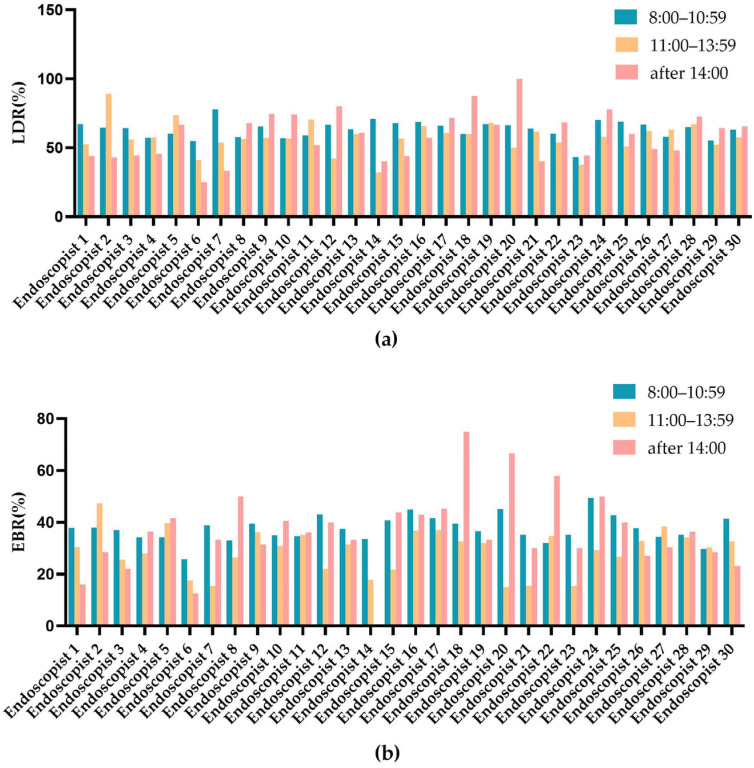
Comparison of individual endoscopist’s LDR and EBR in three shifts. (**a**) The comparison of individual endoscopist’ LDR; (**b**) The comparison of individual endoscopist’ EBR. LDR, lesion detection rate; EBR, endoscopy biopsy rate.

**Figure 4:**
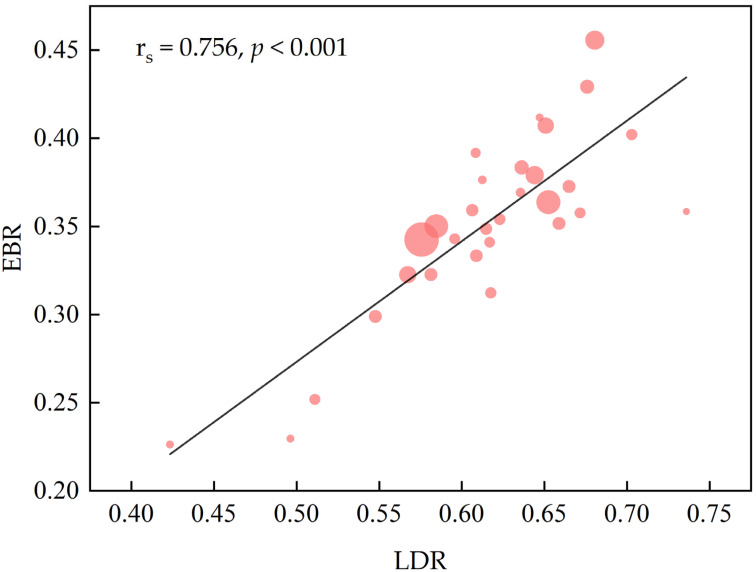
The association between EBR and LDR. Each circle represents a single endoscopist’s performance and the diameter of the circle represents the number of esophagogastroduodenoscopies performed by the endoscopist. LDR, lesion detection rate; EBR, endoscopy biopsy rate; rs, Spearman correlation.

We estimated the individual LDR and EBR for each endoscopist in the study. The comparison of the LDR and EBR of an individual endoscopist across three shifts showed that, for the majority of our endoscopists, the LDR and EBR in shift 2 were lower than those in shift 1 and shift 3 (Figure 3). The LDR range of 30 endoscopists varied between 43.3% and 73.6%, and the EBR varied between 22.6% and 45.6%. The EBR was strongly correlated with the LDR (rs = 0.756, *p* < 0.001) (Figure 4).

### 4.3. Univariate and Multivariate Logistic Regression Analysis

The endoscopy procedures after 14:00 were not performed by the same group of endoscopists than in shift 1 and shift 2. A multivariate regression analysis was conducted, in which the cases in shift 3 were removed. In the multivariate logistic regression analysis controlling for the patients’ gender, age, and conscious sedation, and the endoscopists’ gender (Table 3 and Table 4), we found that the LDR and EBR were significantly associated with the start time. Later procedures were related to lower LDR (OR = 0.965; 95% CI: 0.939–0.996; *p* = 0.012) and lower EBR (OR = 0.930; 95% CI: 0.904–0.956; *p* < 0.001). Other variables, including female patients (*p* < 0.001), patients’ age (*p* < 0.001), conscious sedation (*p* < 0.001), and female endoscopists (*p* < 0.001), were also independent prognostic factors of the LDR and EBR.

## 5. Discussion

To the best of our knowledge, this is the first study to examine the impact of time variables in esophagogastroduodenoscopy. We observed that the lesion detection and endoscopy biopsy rates were similar in EDGs performed in the first three hours of the morning and afternoon. For the procedures performed in the morning schedule, the LDR and EBR in the first three hours were significantly higher than that of later EGD. Furthermore, when the start time for the morning procedures was defined as a 24 h continuous variable, an earlier start time predicted higher LDR and EBR. The diagnostic yield decreased as the day progressed. Each hour elapsed of the day resulted in a 3.5% decrease in the LDR and a 7.0% decrease in the EBR. The effect of the start time was independent of the other pivotal parameters, including patients’ gender, patients’ age, conscious sedation, and endoscopists’ gender.

As the primary objective of EGD is to recognize and diagnose abnormalities, we chose the LDR and EBR as performance measures. We believed that the LDR directly reflected the quality of UGI mucosa inspection. The adequate inspection of mucosa was associated with better recognition of lesions, which need to be confirmed by biopsy. The EBR was proven to be correlated with the detection of premalignant conditions and interval cancer occurrence in a previous study [12]. Similar to that study, our findings indicated that the EBR was significantly associated with the endoscopic detection of lesions. The endoscopists’ competence to identify lesions and the decision to obtain a biopsy may significantly affect the outcomes of EGD.

Previous endoscopic studies have found that time variables have an impact on endoscopy outcomes [15,18,20,21]. Normally, the start time has been compared as a continuous or binary variable. Chan et al. divided colonoscopies into early-morning cases and later cases. They found that patients undergoing early-morning cases had more polyps detected than later cases. Furthermore, when comparing the start time as a continuous variable, they observed a graded decrease in polyp detection [15]. Modi et al. found that the ADR was significantly higher in morning colonoscopies than in afternoon colonoscopies [19]. In our study, the working hours and workload in the afternoon were significantly less than that in the morning. We divided the start time into three shifts in 3 h intervals, which was more suitable for the disproportionate population in this study. As for gastrointestinal procedures, Mehta et.al. found that the time of ERCP did not affect the cannulation success, procedure completion rates, length of procedures, and adverse events [17]. However, diagnostic colonoscopy and EGD are repetitive processes; therefore, cognitive errors may occur as a result of fatigue. Studies about UGI endoscopy are quite rare. More studies have been conducted on starting time and colonoscopy results. Lurix et al. showed that the ADR was not influenced by the passage of time [22]. They hypothesized that this is possibly because of the limited number of conscious sedations, which may lead to fatigue. Modi et al. found that there were no differences in the ADR between morning and afternoon colonoscopies performed in full-day blocks [16]. In comparison to the aforementioned studies, the workload in our center is much heavier, and the high intensity of the work is more likely to lead to the fatigue of the endoscopists. In addition, the continuous working time is also longer. In our center, the continuous working time is up to 6 h in the morning, and the LDR and EBR of the last three hours are significantly lower than the first three hours. Furthermore, there were no differences between the first three hours in the morning and afternoon procedures. We inferred that morning and afternoon were not the factors influencing the outcomes when EGD was performed in half-day blocks. It is possible that continuous work caused the decrease in the LDR and EBR.

There were certain differences in the patient and endoscopists’ characteristics. We performed multivariable logistic regression analysis to assess the factors associated with the LDR and EBR. Male gender, increasing age, and conscious sedation were associated with higher lesion detection and biopsy delivery, similar to previous studies [23]. Female patients and conscious sedation were higher in shift 2 than in shift 1 and patients’ age was more advanced in shift 1 than in shift 2 and shift 3. However, we do not know the reason for this distribution. The three factors have an impact on the EGD results and may offset each other. However, when the start time was measured as a continuous variable for morning procedures, it remained a significant predictor of the LDR and EBR, even after adjusting for these confounders in the multivariable analysis. Furthermore, our study included only outpatient diagnostic EGD, excluding the possibility of scheduling patients with higher risk factors earlier in the list. Based on the study, we hypothesize that fatigue contributed to the results. Given the prolonged, repetitious, and tedious nature of outpatient EGD, the endoscopist could become distracted and make cognitive errors. Endoscopists might be less attentive and vigilant as the day progresses, leading to decreased EGD quality.

The most significant factor associated with EGD outcomes, female gender, was a patient-related, non-modifiable factor in the multivariable analysis. Nevertheless, conscious sedation, endoscopists, and start time were influential procedure-related or endoscopist-related factors that can be modified. These are factors that can be utilized to improve EGD quality. Previous research has shown that conscious sedation affects endoscopic outcomes [24]. We believe that implementing conscious sedation can comfort patients and improve the acceptability of colonoscopy, which can help EGD procedures to progress and improve EGD quality. Reports about endoscopists’ gender discrepancies are rare. A large retrospective cohort study, including 201 physicians, found that female physicians had higher ADR than male physicians [25]. The reason for this may be that female physicians are more deliberate and meticulous when performing endoscopies. Furthermore, sex-related differences in chromatic sensitivity make it easier for female endoscopists to identify lesions [26,27]. Several solutions may help to modify the decrease. One solution is to reduce the duration of continuous work. Our study demonstrated no difference in the LDR and EBR in the first three hours between morning and afternoon procedures. Nevertheless, the LDR and EBR in the last three hours were significantly lower than that in the first three hours in the morning. Similar to our results, a study using Mayo Clinic Rochester schedules indicated that the PDR did not decline as the day progressed when following a 3 h work pattern [28]. We speculate that a 3 h block was more applicable for our center. Another solution is the assistance of artificial intelligence (AI) systems. Many studies have confirmed the effectiveness and robustness of AI systems in improving endoscopic quality [29]. AI systems can alert endoscopists when encountering abnormalities with an acoustic or visual alarm, improving their concentration and vigilance. AI coupled with the expertise of endoscopists has been reported to increase the accuracy of endoscopic diagnosis [30].

There are several limitations in our study. Although the retrospective design has inherent confounders, the multivariable analysis was used to adjust the confounders and proved the robustness of the declining trend. Moreover, one endoscopist did not realize they would be included in the study when performing EDG, thus avoiding the Hawthorne effect. This was a single center study; multi-center studies are needed to further confirm the results. We did not directly measure the endoscopists’ fatigue but used start time as a surrogate indicator. We only speculate that fatigue contributed to the decrease in the LDR and EBR. There is a lack of evidence that fatigue had a role in these results. The examination time is an important quality indicator of EGD [11,31], but was not recorded in our study. We could not determine if endoscopists will reduce the examination time and lead to a decrease in lesion identification as time progresses and the workload increases. The key strength of this study is its large sample size of diagnostic EGD performed by 30 endoscopists with high procedure volumes.

## 6. Conclusions

In conclusion, our study found that the lesion detection and endoscopy biopsy rates decreased as the day progressed. This was probably due to endoscopist fatigue. The relationship requires more investigation. Shortening the procedure schedule is recommended to improve esophagogastroduodenoscopy quality.

## Figures and Tables

**Table 1:** Comparison of risk factors and outcomes among three time periods.

Time	All Periods (*n* = 14,597)	8:00–10:00 (*n* = 10,208)	11:00–13:00 (*n* = 3541)	After 13:00 (*n* = 848)	*p* Value
Lesions detection	7342 (61.4%)	5161 (62.4%)	1735 (58.5%)	446 (61.0%)	<0.001
Biopsy	5229 (35.8%)	3814 (37.4%)	1114 (31.5%)	301 (35.5%)	<0.001
Patients’ gender, female, *n* (%)	7666 (52.5%)	5337 (52.3%)	1924 (54.3%)	405 (47.8%)	0.002
Patients ‘age, median (25–75%)	49 (30–58)	50 (37–59)	46 (33–57)	44.5 (31–56)	<0.001
Conscious sedation	10,533 (72.2%)	7016 (68.7%)	2944 (83.1%)	573 (67.6%)	<0.001
Endoscopists’ gender, female, *n* (%)	7920 (54.3%)	5346 (52.4%)	2030 (57.3%)	544 (64.2%)	<0.001

**Table 2:** Comparison of EGD findings among three time periods.

Findings	8:00–10:00 (*n* = 10,208)	11:00–13:00 (*n* = 3541)	After 13:00 (*n* = 781)	*p* Value
Barrett’s esophagus	329 (3.47%)	0 (0.00%)	25 (3.20%)	<0.001
Submucosal lesion	1882 (21.26%)	169 (4.77%)	119 (15.24%)	<0.001
Polyp	2416 (30.88%)	439 (12.40%)	297 (38.03%)	<0.001
Intestinal metaplasia	226 (3.06%)	70 (1.98%)	16 (2.05%)	0.69
Ulcer	1120 (15.75%)	368 (10.39%)	120 (15.36%)	<0.001
H.pylori infection	296 (4.37%)	115 (3.25%)	35 (4.48%)	0.037
Cancer	73 (1.06%)	9 (0.25%)	26 (3.33%)	<0.001
Reflux esophagitis	1114 (11.99%)	1 (0.03%)	109 (13.96%)	<0.001
Diverticulum	70 (0.84%)	10 (0.28%)	6 (0.77%)	0.021
Bile reflux	591 (6.63%)	58 (1.64%)	28 (3.59%)	<0.001

**Table 3:** Univariable logistic regression analysis.

Outcome	LDR			EBR		
	OR	95% CI	*p* Value	OR	95% CI	*p* Value
Patients’ gender (Female)	0.778	0.726–0.833	0.001	0.801	0.747–0.859	<0.001
Patients’ age	1.040	1.038–1.043	<0.001	1.033	1.030–1.035	<0.001
Conscious sedation	1.269	1.176–1.370	<0.001	1.33	1.228–1.440	<0.001
Endoscopists’ gender (Female)	1.233	1.151–1.321	<0.001	1.282	1.195–11.375	<0.001
Time of the day	0.931	0.907–0.956	<0.001	0.910	0.886–0.935	<0.001

LDR, lesion detection rate; EBR, endoscopy biopsy rate.

**Table 4:** Multivariable logistic regression analysis.

Outcome	LDR			EBR		
	OR	95% CI	*p* Value	OR	95% CI	*p* Value
Patients’ gender (Female)	0.701	0.652–0.754	<0.001	0.737	0.686–0.792	<0.001
Patients’ age	1.041	1.038–1.044	<0.001	1.033	1.030–1.036	<0.001
Conscious sedation	1.288	1.188–1.396	<0.001	1.388	1.277–1.508	<0.001
Endoscopists’ gender (Female)	1.268	1.180–1.362	<0.001	1.312	1.221–1.411	<0.001
Time of the day	0.965	0.939–0.996	0.012	0.930	0.904–0.956	<0.001

LDR, lesion detection rate; EBR, endoscopy biopsy rate.

## Data Availability

Not applicable.
